# Systematic analysis of the cuprotosis in tumor microenvironment and prognosis of gastric cancer

**DOI:** 10.1016/j.heliyon.2023.e13831

**Published:** 2023-02-17

**Authors:** Ke-wei Wang, Mei-dan Wang, Zi-xi Li, Ben-shun Hu, Jian-feng Huang, Jun-jie Wu, Zheng-dong Yuan, Xiao-long Wu, Qin-fang Yuan, Yi-fan Sun, Feng-lai Yuan

**Affiliations:** a Institute of Integrated Traditional Chinese and Western Medicine, Affiliated Hospital of Jiangnan University, Wuxi, China; b Department of Hepatobiliary Surgery, Affiliated Hospital of Jiangnan University, Wuxi, China; c Department of Radiation Oncology, Affiliated Hospital of Jiangnan University, Wuxi, China; d Department of Hospital Infection, Affiliated Hospital of Jiangnan University, Wuxi, China; e Clinical Laboratory, Affiliated Hospital of Jiangnan University, Wuxi, China

**Keywords:** Cuprotosis, Tumor microenvironment, Prognosis, Gastric cancer

## Abstract

Cuprotosis is a new programmed cell death related to cancer. However, the characteristics of cuprotosis in gastric cancer (GC) remain unknown. Ten cuprotosis molecules from 1544 GC patients were used to identify three GC molecular genotypes. Cluster A was characterized by the best clinical outcome and was significantly enriched in metabolic signaling pathways. Cluster B exhibited elevated immune activation, high immune stroma scores and was significantly enriched in tumor immune signaling pathways. Cluster C was characterized by severe immunosuppression and poor response to immunotherapy. Notably, the citrate cycle, cell cycle, and p53 signaling pathways were enriched in the differentially expressed genes among the three subtypes, which were critical signaling pathways for cell death. We also developed a cuprotosis signature risk score that could accurately predict the survival, immunity, and subtype of GC. This study presents a systematic analysis of cuprotosis molecules and provides new immunotherapeutic targets for GC patients.

## 1 Introduction

Gastric cancer (GC) ranks fifth and fourth in terms of morbidity and mortality, respectively, among all malignant tumours [1]. GC is difficult to diagnose in its early stages since its symptoms are nonspecific or unreliable. Although there has been notable progress in GC treatment, including surgical resection, gene therapy, radiotherapy and chemotherapy, the five-year survival for patients with advanced GC is still below 40% [2]. Therefore, it is necessary to assess the prognosis of GC patients using biomarkers related to GC prognosis at an early stage.

Copper ions are present in organisms, but their concentration is kept low and their balance is constantly shifting. Cuprotosis, the death of cells caused by copper toxicity, is distinct from apoptosis, necrosis, and iron death [3,4]. Direct copper ion binding to lipoacylated tricarboxylic acid cycle components causes aberrant aggregation of lipoacylated proteins and loss of iron-sulphur cluster proteins, leading to toxic protein stress and ultimately mediating cell death. Many types of cancer, including breast, thyroid, cervical, ovarian, lung, pancreatic, prostate, oral, and bladder cancer, have been associated to considerable alterations in copper level in serum and tumor tissues, according to previous studies [[5], [6], [7], [8], [9], [10]]. Copper may play a key role in the cause, severity and progression of cancer. For example, copper can promote angiogenesis in tumours by activating many angiogenic factors, including angiopoietin, vascular endothelial growth factor, fibroblast growth factor 1 and interleukin 1 [[11], [12], [13]].

In both clear cell renal cell carcinoma and head and neck squamous cell carcinoma (HNSC), cuprotosis has been related to a rise in immune cell infiltration in recent years [14,15]. It is widely known that inflammatory indicators have essential implications for tumor immunotherapy and prognosis. Several studies demonstrate that inflammatory markers derived from hemograms, such as erythrocyte distribution width, mean platelet volume, and platelet/lymphocyte ratio, are significantly altered in the blood and tumor tissues of patients with thyroid cancer [[16], [17], [18]]. Next, it has been established that patients with stomach cancer and healthy individuals have significantly different serum copper levels [19]. In practice, patients in the 4th quartile have a 2.42-fold higher risk of stomach cancer than those in the 1st quartile [20]. Additionally, a copper complex (Copper–Zinc Superoxide Dismutase, odds ratio = 3.01) is associated with a higher stomach cancer incidence [21]. Therefore, we proposed that copper ions might contribute to the onset of gastric cancer by altering cellular changes and the immune microenvironment. It was required to further assess whether the cuprotosis was a possible target for gastric cancer prevention and survival prediction.

This study used a panel of 10 cuprotosis molecules to divide 1544 GC patients into three distinct subgroups (Cluster A, B and C). Prognosis and immune cell infiltration were compared among the three subtypes. In addition, a risk score consisting of four cuprotosis molecules was constructed to quantify patients’ cuprotosis levels. Finally, we selected hub genes to understand immune infiltration and immunotherapy using multi-color immunofluorescence staining. The study suggests that GC subtypes and risk scores in relation to cuprotosis molecules may be able to provide a reference for the individualized treatment and evaluation of GC patients.

## 2 Methods

### 2.1 Patients and datasets

Our literature search using multiple databases identified GC gene-expression datasets and corresponding survival outcomes or immunotherapy. This study thus included eight GC cohorts: GSE15459, GSE29272, GSE34942, GSE57303, GSE62254, GSE84437, Kim cohort and TCGA-STAD cohort. Six Gene-Expression Omnibus (GEO) cohorts came from the GEO database (https://www.ncbi.nlm.nih.gov/), the TCGA-STAD cohort from the TCGA set (https://portal.gdc.cancer.gov/) and the Kim cohort from a previous study [22]. Information on demographics and clinical factors was culled from the websites and relevant articles. From the eight datasets, we gained 1748 patients, of whom 1544 patients with survival information comprised a merged cohort (MC), 45 patients with immunotherapy comprised the immunotherapy cohort, and 156 patients without survival or immunotherapy were excluded.

During the period of January 2019 through December 2020, 25 GC patients from the clinical dataset had gastrectomy in the affiliated hospital of Jiangnan University, Wuxi 214,122, China. Twenty-five samples preserved in formalin and paraffin were chosen for the clinical dataset. All subjects provided their written informed consent to participate. The study was conducted in accordance with the principles outlined in the Declaration of Helsinki and with the approval of the ethics committee of the affiliated hospital of Jiangnan University.

Four subtypes of GC – epithelial-to-mesenchymal transition (EMT), microsatellite instability (MSI), intact TP53 activity (MSS/TP53^+^) and TP53 functional loss (MSS/TP53^−^) – were provided by Cristescu R et al. [22]. from the Department of Genetics and Pharmacogenomics, Merck Research Laboratories, Merck Sharp & Dohme, Boston, Massachusetts, USA.

The SVA package in R [24] was used to perform a log2 (x + 1) transformation and batch rectification on all gene expression or transcriptome data. Data on GC immunotherapy from the Kim cohort included RNAseq results (PRJEB25780, https://www.ebi.ac.uk/ena/data/view/PRJEB25780), ICI response, TCGA subtype, microsatellite instability (MSI), Epstein-Barr virus (EBV), mesenchymal subtype, single nucleotide variants (SNVs), and immunological signature [22]. Immunotherapy response was defined in the study as either a partial response (PR) or a complete response (CR). Individuals with stable disease (SD) and those with progressive disease (PD) were among those who did not respond to immunotherapy.

### 2.2 Gene set genomic analyses of 10 cuprotosis molecules in pan-cancer from TCGA

We used Gene Set Cancer Analysis (GSCA; http://bioinfo.life.hust.edu.cn/GSCA/#/) to perform gene set genomic analyses, including gene differential expression, overall survival (OS), SNV, copy number variation (CNV), methylation, pathway activity, miRNA regulation and normal tissue expression across 33 cancer types from TCGA. GSCA consists of multi-omics data from 11,160 samples across 33 cancer types (TCGA Cancer).

According to the GSCA, the mRNA expression data and Illumina Methylation 450 k level 3 data of the 10 genes associated with cuprotosis were downloaded from the TCGA database and merged with the TCGA barcode. The TCGA database was also mined for SNV information from 10,234 patients across 33 different cancer types.

### 2.3 Clustering of 10 cuprotosis molecules identified by unsupervised learning

Unsupervised clustering of expression data or transcription data of the 10 cuprotosis genes from 1544 patients using the ConsensuClusterPlus R package was utilized to determine the classifications of three clusters [25,26]. To guarantee the reliability of the classification, this procedure was carried out a total of a thousand times.

### 2.4 Assessment of the immune microenvironment

Some indicators of the immune microenvironment, including immune cell invasions, stromal cell populations and immune function scores, were assessed by MCPCOUNTER, QUANTISEQ, CIBERSORT, XCELL, TIMER and EPIC [[27], [28], [29], [30], [31], [32], [33]]. The TIDE web server was used to predict the immunotherapy response based on standardised expressions of genes [31].

### 2.5 Analysis of the biological function and pathways of genes

The molecular function, cellular component, and biological process annotations of genes were done using the Gene Ontology (GO) database and informatics resource (http://www.geneontology.org). Enrichment pathways of genes were annotated using data from the Kyoto Encyclopedia of Genes and Genomes (KEGG) database (http://www.genome.ad.jpl/kegg/), which offers studies of the senior functional behaviors of cells and organisms. Signaling pathways and molecular functions were identified using gene set enrichment analysis (GSEA) or single sample GSEA (ssGSEA) [34,35]. The GSCALite was also used to find the major pathways involved in hub genes [36].

### 2.6 Construction of cuprotosis signature for prognosis

Cox regression in the MC and logistic regression in the Kim cohort were used to develop a prediction model for prognosis and immunotherapy response, respectively, using ten cuprotosis molecules. Cox or logistic regression coefficients and gene expression data were used to determine the risk score of the predictive model. Here's the exact equation: $risk\;score=\sum \left(expression\;of\;{gene}_{i}\times {coefficient}_{i}\right)$. According to the cutoff thresholds derived using the Youden index, the risk scores of samples being continuous variables were regrouped into dichotomous variables (high-risk group and low-risk group). Kaplan-Meier (KM) and Cox regression analyses were used to analyze the survival of GC patients in different risk categories in order to evaluate the prognostic value of the cuprotosis signature. The R's rms or survival tool was used to run the Cox regression, and R's regplot package was used to generate the nomogram [37]. Receiver operating characteristics (ROC) curves for three-, five-, and eight-year survival were generated using the timeROC package of R [38]. R's rms package and rmda package were used to plot the calibration curves and decision curves of the nomogram.

### 2.7 Specimen collection

Twenty-five GC patients who had surgery in the affiliated hospital of Jiangnan University provided samples. Preoperative treatment included neither chemo nor radio nor biotherapy. All participants provided written informed consent, and the study was conducted in accordance with the principles outlined in the Declaration of Helsinki and with the approval of the ethics committee of the affiliated hospital of Jiangnan University. Two separate pathologists confirmed the GC diagnosis in all specimens. For immunohistochemical staining, 4 μm slices were cut from all specimens that had been fixed in formalin and embedded in FFPE.

### 2.8 Haematoxylin–eosin staining (HE)

Dewaxing was done using xylene on the FFPE sample. Afterward, gradient alcohol was used to deoxidize the xylene. Using haematoxylin, we stained the tissue for 3 min. Under the microscope, we could see staining of the nuclei. To further evaluate the staining, the tissue was incubated with the eosin solution for 90 s before being viewed under a microscope.

### 2.9 Immunohistochemistry (IHC)

After dewaxing and hydrating the FFPE, the EDTA technique was used to restore the tissue antigen. Tissue peroxidase was rendered inactive using hydrogen peroxide. The tissue was then immersed in primary antibody solution and kept in a wet box at 4° Celsius for 12 h. After that, the tissue sat at room temperature for half an hour to rewarm. After adding a second antibody labeled with horseradish peroxidase and letting it incubate for 60 min at room temperature, the tetramethylbenzidine color was seen. The favorable color reaction of the brownish-yellow particles was seen with an optical microscope.

This research made use of the following primary antibodies: CK (ab52625, Abcam), FDX1 (NBP1-89227, Novus), PDHA1 (ab168379, Abcam), PD-L1(ab205921, Abcam) and CD8 (14-0081-82, Invitrogen Antibodies). The breadth and number of tissues infiltrated by CD8^+^ T lymphocytes were used to establish immune classifications of GC [39,40]. Tumor parenchyma and stroma were infiltrated by CD8^+^ T lymphocytes, a hallmark of the immune-inflamed subtype. CD8^+^ T cells infiltration was found exclusively in the peritumour stroma, but not in the parenchyma, marking the excluded immune subtype. The lack of CD8^+^ T cells in tumor parenchyma and stroma was a hallmark of the deserted immune subtype.

### 2.10 Multi-colour immunofluorescence

The FFPE section was dewaxed, and then the EDTA antigen repair buffer was applied to it (PH 9.0). After adding phosphate-buffered saline (pH7.4) and the primary antibody, the tissues were left in a refrigerator at 4° Celsius for 12 h. After 50 min of room-temperature incubation, the tissues were stained with secondary antibodies. After adding DAPI dye, the mixture was incubated for 10 min at room temperature, out of the light. After a quick shaking, the slices were vacuum-sealed with tablets that extinguish fluorescence. The distribution, conformation, and abundance of the protein were all photographed using a fluorescence microscope.

### 2.11 Statistical analysis

In this study, the following statistical methods were used: Shapiro-Wilk test for normality analysis of continuous variables; independent *t*-test or F-test for comparison of continuous variables with normal distributions; univariate and multivariate Cox regression for screening factors affecting survival; the Wilcoxon test for comparison of risk scores in different groups; ROC analysis for evaluating the effect of statistical models predicting prognosis of GC patients; the Spearman correlation analysis for continuous variables; and the Chi-square test for categorical variables. The Youden index derived the cut-off thresholds for the cuprotosis signature model. A two-sided p-value <0.05 was considered significant. All statistical analyses were performed using R software version 4.0.3.

## 3 Results

### 3.1 The profiles of 10 cuprotosis molecules in pan-cancer from TCGA

**Fig. 1:**
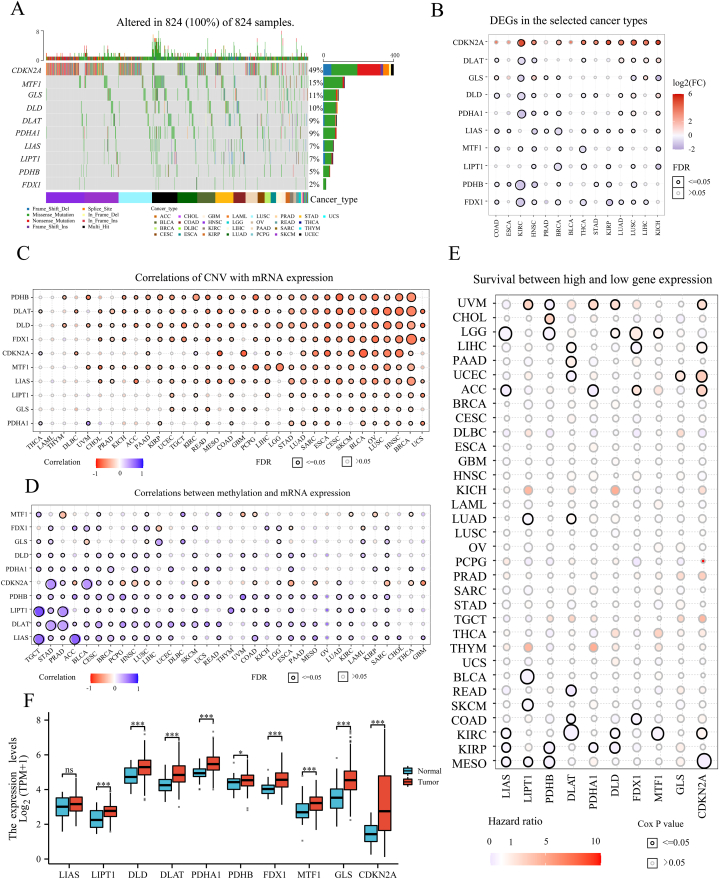
**Profiles of 10 cuprotosis molecules in pan-cancer database. A** Mutation of 10 cuprotosis moleculs in pan-cancer tissues. **B** The expression of 10 cuprotosis genes in paired Tumor and normal samples. DEGs: differentially expressed genes between paired Tumor and normal tissues across 33 cancer types (TCGA Cancer).TCGA: The Cancer Genome Atlas. The genes with fold change (FC) > 2 and significance with false discovery rate (FDR) ≤0.05. **C** Correlations between copy number variation (CNV) and mRNA expression level in pan-cancer tissues. Spearman correlation analysis was performed. **D** Correlations between methylation and mRNA expression levels in pan-cancer tissues. Spearman correlation analysis was performed. **E** Association between 10 cuprotosis genes and survival in pan-cancer tissues. Median mRNA value was used to divide tumor samples into high and low expression groups. Cox proportional-hazards model and Log-rank tests were performed for every gene in every cancer. **F** Comparison of 10 cuprotosis genes expression between Tumor and normal tissues in TCGA-STAD. TPM: transcripts per million; STAD: stomach adenocarcinoma; *p < 0.05; **p < 0.01; ***p < 0.001.

Ten cuprotosis molecules, including CDKN2A, DLAT, DLD, FDX1, GLS, LIAS, LIPT1, MTF1, PDHA1 and PDHB, have been found in earlier research [4]. This study used the GSCA to perform SNVs across 33 cancer types from TCGA. Only 824 patients were found to have the mutation of the 10 cuprotosis genes, and CDKN2A mutation had the highest frequency (49%), followed by MTF1 (15%) and GLS (11%) (Fig. 1A). To explore the differential expression of 10 cuprotosis genes in paired tumour and normal tissues across 33 cancer types (TCGA Cancer), GSCA only selected cancer types with more than 10 paired tumours and normal tumour type samples and finally found differential expression of 10 genes in 14 cancer types, including bladder urothelial carcinoma (BLCA), breast invasive carcinoma (BRCA), colon adenocarcinoma (COAD), oesophageal carcinoma (ESCA), head and neck squamous cell carcinoma (HNSC), kidney chromophobe (KICH), kidney renal clear cell carcinoma (KIRC), kidney renal papillary cell carcinoma (KIRP), liver hepatocellular carcinoma (LIHC), lung adenocarcinoma (LUAD), lung squamous cell carcinoma (LUSC), prostate adenocarcinoma (PRAD), stomach adenocarcinoma (STAD) and thyroid carcinoma (THCA) (Fig. 1B). Genetic variation is an essential factor affecting the expression of cuprotosis molecules. We found that CNV and mRNA expression levels of cuprotosis molecules were inversely correlated in most cancer types (Fig. 1C). Moreover, the high frequency of heterozygous amplification and heterozygous deletion of the 10 cuprotosis molecules were ubiquitous in most cancer types, while the low frequency of homozygous amplification and homozygous deletion were ubiquitous in most cancer types (Figs. S1A and B). Most cancer types have more than three types of CNV for each cuprotosis molecule (Fig. S1C). Similarly, significant negative correlations between methylation and mRNA expression of the 10 genes were found in most cancer types (Fig. 1D). Significant methylation differences of the 10 cuprotosis molecules between paired tumour and adjacent non-tumour samples were observed in 14 cancer types (Fig. S1D).

Next, samples with mRNA values exceeding the median value were assigned to the high expression group, while others were assigned to the low expression group. The 10 cuprotosis molecules were related to the prognosis of uveal melanoma (UVM), cholangiocarcinoma (CHOL), brain lower grade glioma (LGG), LIHC, COAD, KIRC, KIRP and mesothelioma (MESO) (Fig. 1E). Although the 10 cuprotosis molecules were not significantly associated with the survival of samples from the TGCA-STAD cohort, nine genes showed highly significant differential expression in tumor versus normal tissues (Fig. 1F). Furthermore, differential mRNA expressions of the 10 cuprotosis molecules were observed among subtypes of specific cancers including HNSC, LUSC, COAD, STAD, LUAD, glioblastoma multiforme (GBM), BRCA, KIRC and BLCA (Fig. S1E).

### 3.2 Three subtypes of GC according to 10 cuprotosis molecules identified by unsupervised learning

**Fig. 2:**
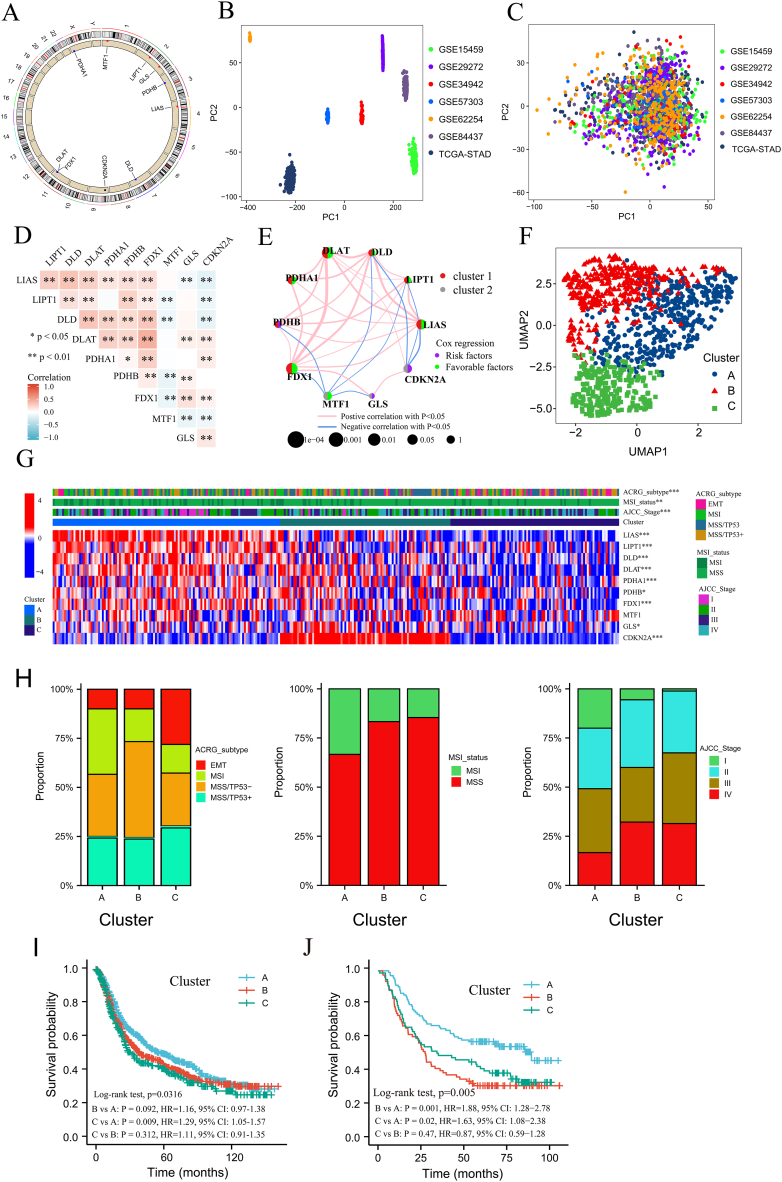
**The subtypes of GC were classified by 10 cuprotosis molecules. A** Distribution of 10 cuprotosis genes on 23 chromosomes and regulation of CNV in TCGA-STAD. The gene expression change throughout eight cohorts before (**B**) and after batch effect correction (**C**). **D** The heat map of interrelations between 10 cuprotosis molecules. Spearman correlation analysis was performed. **E** Interrelations and prognosis of 10 cuprotosis molecules in GC. Spearman correlation analysis was performed. **F** uniform manifold approximation and projection (UMAP) showed the classification of 10 cuprotosis molecules after unsupervised learning clustering of 1544 patients. **G** The relationship between three subtypes of GC and 10 cuprotosis genes and other subtypes in the GSE62254 cohort. *p < 0.05; **p < 0.01; ***p < 0.001. H Samples from three subtypes of GC distributed in ACRG, MSI status and AJCC Stage subtypes. Effects of the three subtypes of GC on the survival of individuals in the MC (I) and GSE62254 cohort (J). HR: hazard ratio; CI: confidence interval.

We found that five cuprotosis molecules, DLAT, PDHA1, FDX1, GLS and CDKN2A, were significantly different between paired tumour and adjacent non-tumour samples from the TCGA-STAD cohort (Fig. S2A), and LIAS, DLD, DLAT and MTF1 were significantly different among the four pathologic T categories 1–4 (Fig. S2B). The distribution of the 10 cuprotosis molecules on 23 chromosomes and the regulation of CNV are shown in Fig. 2A. Of the cuprotosis molecules, CDKN2A had the highest altered frequency (32%), followed by DLD (19%) and MTF1 (16%; Fig. S2C).

To explore the effect of the cuprotosis molecules on the survival of GC in a relatively large sample size, 1544 patients were chosen from the GSE15459, GSE29272, GSE34942, GSE57303, GSE62254, GSE84437, Kim and TCGA-STAD cohorts, comprising the MC. This cohort was found to be dispersive using principal component analysis (PCA) (Fig. 2B) and was made uniform after batch correction (Fig. 2C). Of the 10 cuprotosis molecules, any two having significant correlations were common (Fig. 2D), and four – LIAS, DLAT, PDHA1 and FDX1 – were favorable factors of prognosis in GC (Fig. 2E and Table S1). By using unsupervised hierarchical clustering with 10 cuprotosis molecules on 1544 GC patient data, we were able to categorize the GC samples into three distinct categories (Cluster A, Cluster B and Cluster C, Figs. S2D–K). Dimension reduction analysis of high-dimensional data using uniform manifold approximation and projection (UMAP, Fig. 2F) and PCA (Fig. S2L) validated three subtypes (cuprotosis subtypes) of GC according to the 10 cuprotosis molecules.

Next, we found significant interrelationships between cuprotosis subtypes and the other three subtypes, including the Asian Cancer Research Group (ACRG) subtype, MSI status and ACRG subtype, in the GSE62254 cohort (Fig. 2G). Cluster C had a high proportion of the EMT subtype ($\chi^{2}$ = 30.9, p < 0.0001), MSS subtype ($\chi^{2}$ = 12.9, p = 0.002), AJCC Stage III and IV subtype ($\chi^{2}$ = 28.3, p < 0.0001), which had poor survival (Fig. 2H). There were nine cuprotosis molecules with significant differences among the three cuprotosis subtypes (Fig. 2G and Fig. S3A). Kaplan-Meier survival curves showed significantly different survival rates among three cuprotosis subtypes from the MC (Fig. 2I) and the GSE62254 cohort (Fig. 2J and Fig. S3B), and Cluster A had the best survival.

### 3.3 The tumour microenvironment (TME) of the three cuprotosis subtypes

**Fig. 3:**
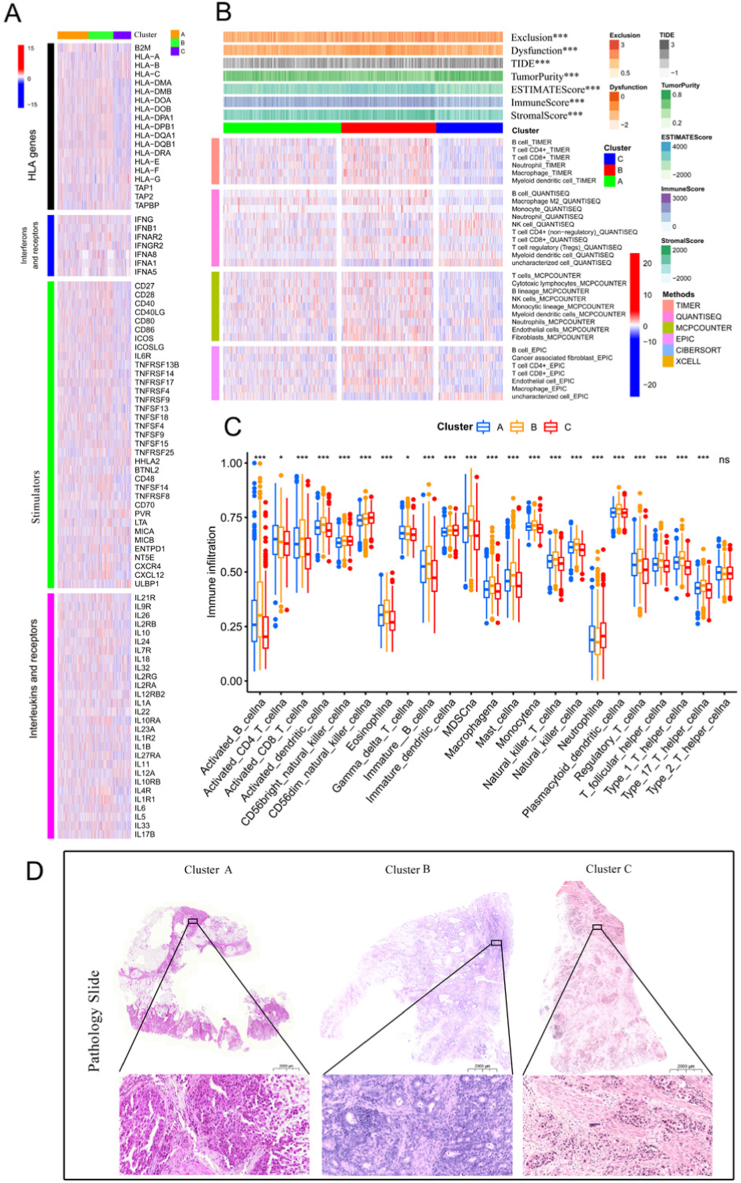
**Immune microenvironment in three cuprotosis subtypes. A** Expressions of HLA, interferons and receptor, stimulator, interleukin and receptor genes in Clusters A, B and C. **B** Distribution of immune cell infiltration, stromal tissue scores, immune function scores and immune escape scores in Clusters A, B and C. **C** Comparison of the single-sample gene set enrichment analysis (ssGSEA) scores in Clusters A, B and C. **D** Representative haematoxylin-eosin (HE) pathology staining diagram in Clusters A, B and C (A: TCGA-CG-4444-01A-01R-1157-13; B: TCGA-CG-5719-01A-11R-1602-13, C: TCGA-HU-A4GC-11A-11R-A251-31; TCGA-STAD database).

Many genes of immune-related functions, such as human leukocyte antigen (HLA), interferons (IFN), stimulators, interleukins, inhibitors and chemokines, play essential roles in regulating tumour cells via the immune system. Of 148 immune-related genes, 114 gene expressions differed significantly among the three cuprotosis subtypes (Fig. 3A, Fig. S4A and Table S2). Among 19 HLA genes, four genes B2M, HLA-A, HLA-E and HLA-G which belonged to MHC I showed significant differences among three cuprotosis subtypes [41,42] (all <0.05, Fig. 3A and Table S2). Moreover, the expressions of stimulator genes were higher in Clusters A and B (Fig. 3A), and the interleukin genes were more expressed in Cluster B. Only four interferon genes, IFNGR2, IFNA8, IFNAR2 and IFNG, had significant differences among the three cuprotosis subtypes. Most inhibitor genes had higher expressions in Cluster C, while chemokines and receptor genes had higher expressions in Clusters A and B. Immune cell infiltration, immune stroma score, immune function score and immunotherapy response were assessed by MCPCOUNTER, QUANTISEQ, CIBERSORT, XCELL, TIMER, EPIC and TIDE. It was seen that B cell, CD8 T cell, CD4 T cell, Tregs and M2 macrophages were more prevalent in Cluster B; whereas, M0 macrophages, activated dendritic cells, Th1 cells, Th2 cells and natural killer T cells were more prevalent in Cluster C (Fig. 3B, Fig. S4B and Table S3).

The ssGSEA was used to assess the relative abundance of each cell's infiltration in tumour tissues and showed that activated B cell, activated CD8 T cell, activated dendritic cell, immature B cell, macrophage, mast cell, monocyte, natural killer T cell, plasmacytoid dendritic cell, regulatory T cell, T follicular helper cell, Type 1 T helper cell, Type 17 T helper cell were the highest in the Cluster B (Fig. 3C). Additionally, Cluster B had the highest stromal score, immune score, ESTIMATEScore, dysfunction score and exclusion score, while Cluster C had the highest tumour purity score and TIDE (Fig. 3B and Table S3). These results suggested that Cluster B had a higher immune infiltration status than Clusters C and A. The TCGA pathology slide also verified that Cluster B tumours had more abundant immune cells (Fig. 3D). Several essential immune functions, including immune checkpoint, cytolytic activity, HLA, inflammation promoting, Type II IFN response and CCR had the highest scores in Cluster B and the lowest scores in Cluster C (Fig. S4C).

### 3.4 Potential functional role of the three cuprotosis subtypes

**Fig. 4:**
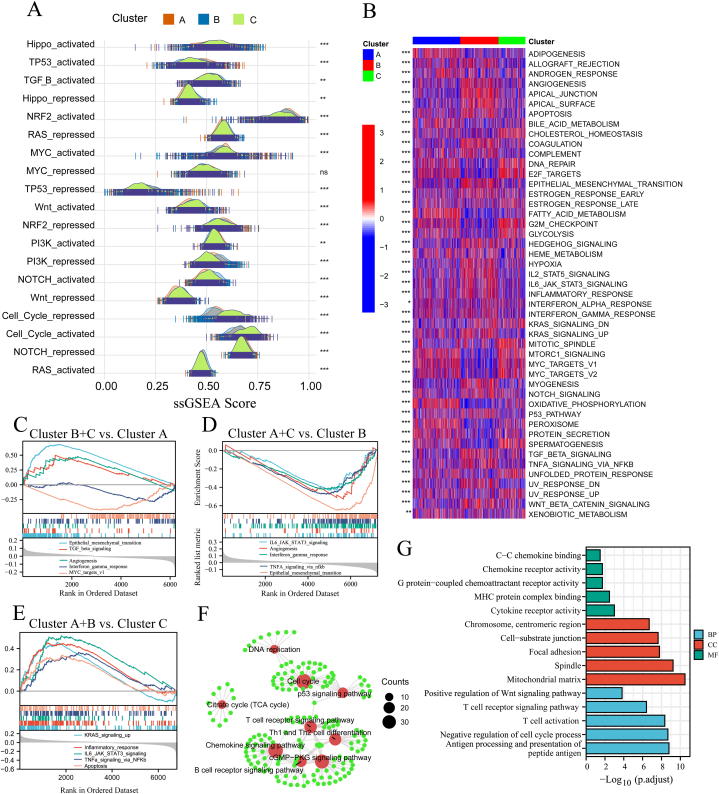
**Important functions and pathways enriched in three cuprotosis subtypes. A** Crucial pathways of gastric carcinogenesis using the single sample gene set enrichment analysis (ssGSEA) in Clusters A, B and C. **B** 40 signaling pathways using gene set enrichment analysis (GSEA) in Clusters A, B and C. **C-E** Comparison of particular biological pathways using GSEA in Clusters A. B and C. Kyoto encyclopedia of genes and genomes (KEGG) enrichment analysis (**F**) and Gene Ontology (GO) enrichment analysis (**G**) of differentially expressed genes among Clusters A. B and C.

The enrichment scores of several carcinogen pathways showed that Custer B was characterised by RAS, NOTCH, MYC, TGF−B and Hippo activation and by Wnt, PI3K, RAS and Hippo repression (Fig. 4A). TP53, NRF2 and MYC activation and NRF2 and NOTCH repression were found in Cluster A, while Custer C was characterised by the cell cycle pathway (Fig. 4A). Furthermore, GSVA was used to quantify the enriched pathways of differentially expressed genes among the three cuprotosis subtypes of GC. Among the 47 pathways that showed significant differences among the three cuprotosis subtypes, 24 pathways had the highest scores in Cluster B. These included interferon gamma response, interferon alpha response, inflammatory response, IL6-JAK-STAT3 signaling, IL2-STAT5 signaling, EMT, apoptosis, and so on (Fig. 4B). Several metabolic pathways such as adipogenesis, androgen response, bile acid metabolism, and fatty acid metabolism had the highest scores in Cluster A. Finally, DNA repair, KRAS signalling DN, late oestrogen response and G2M checkpoint had the highest scores in Cluster C (Fig. 4B and Table S4). Similar gene set enrichment results were observed in Clusters A, B and C (Fig. 4 C–F and Tables S5–7).

Among the three cuprotosis GC subtypes, 1735 differentially expressed genes were shared between any two groups (FDR< 0.001) (Fig. S5A and Table S8), and KEGG analysis revealed that these genes were enriched in critical signaling pathways, including T cell receptor signalling pathway, B cell receptor signalling pathway, Th1 and Th2 cell differentiation and chemokine signalling pathway (Fig. 4F and Table S9). GO enrichment analysis showed that the 1735 differentially expressed genes were mainly enriched in immune functions such as C–C chemokine binding, chemokine receptor activity, G protein-coupled chemoattractant receptor activity, MHC protein complex binding, cytokine receptor activity in the MC, and tumour immunities, including positive regulation of the Wnt signalling pathway, T cell receptor signalling pathway, T cell activation, negative regulation of cell cycle process and antigen processing and presentation in BP (Fig. 4G and Table S10). Next, we used the GSCALite to assess the effect of the 10 cuprotosis genes on several tumorigenic pathways and found that DLAT, FDX1, DLD, PDHA1 and CDKN2A could activate the AR hormone, apoptosis and cell cycle in GC, and inhibit RAS/MAPK and EMT (Fig. S5B) [36]. The correlation calculated for all paired samples (33 cancer types) revealed that of the 10 cuprotosis molecules, six (DLAT, DLD, MTF1, CDKN2A, GLS and FDX1) may be negatively regulated by many miRNAs (Fig. S5C).

### 3.5 Construction of the cuprotosis signature

**Fig. 5:**
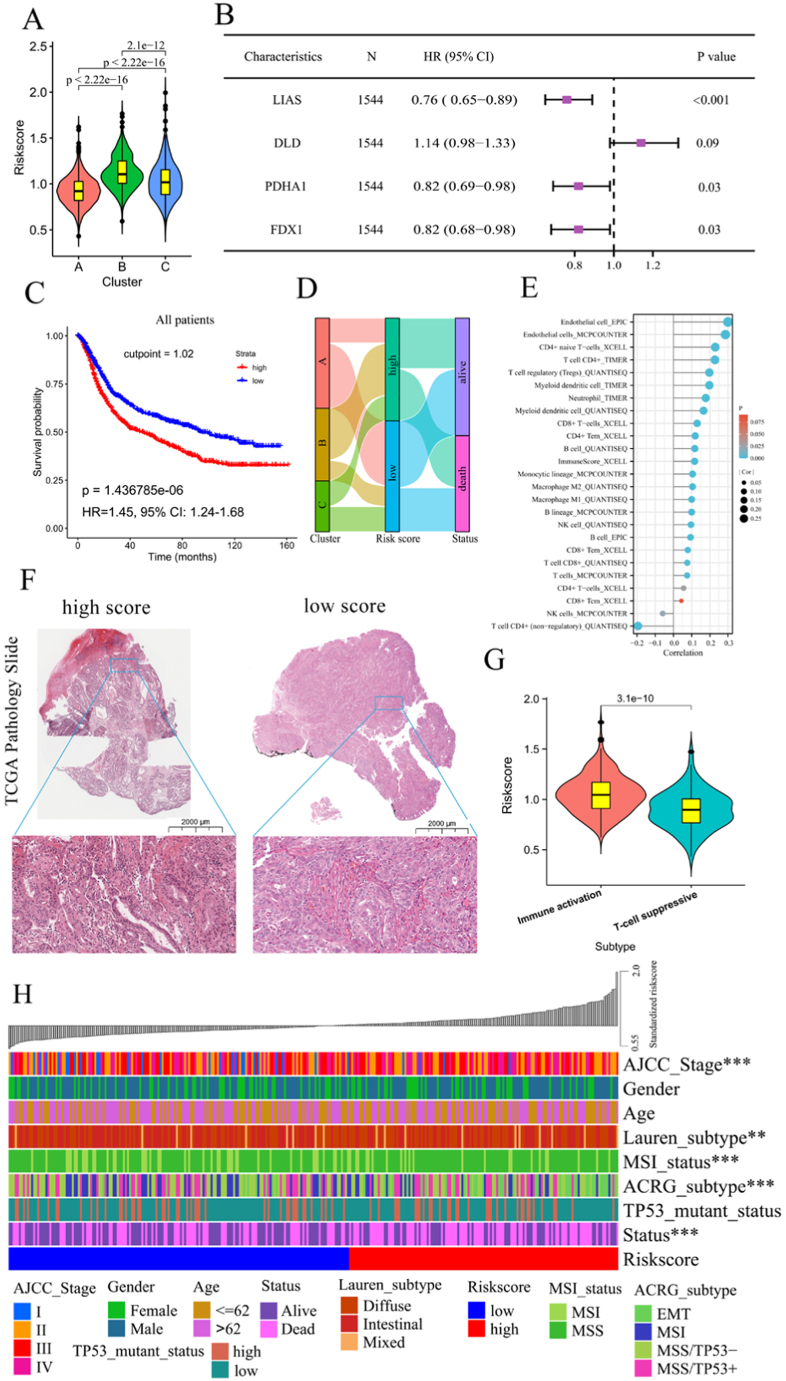
**Association between the risk scores of the cuprotosis signature and immune microenvironment and clinical features. A** Comparison of the risk scores among three cuprotosis subtypes of GC. **B** Risk prediction model (the cuprotosis signature) established by multivariate Cox regression. **C** Kaplan-Meier curves of both groups' survival in the MC. **D** Sankey diagram showed the relationship between the risk scores of the cuprotosis signature and three cuprotosis subtypes and the prognosis of GC. **E** Correlations between risk scores and immune cell infiltration. Spearman correlation analysis was performed. **F** Representative HE pathology staining diagram in both groups (high-group: TCGA-HU-8610-01A-22R-2402-13; low-group: TCGA-FP-8211-01A-11R-2343-13; TCGA-STAD database). **G** Comparison of risk scores in two subtypes of GC, including immune activation and T-cell suppressive groups. **H** Relationships between risk scores and other clinical features in the GSE62554 cohort.

We proposed a cuprotosis signature model using backward stepwise multivariate Cox regression with the 10 candidate cuprotosis molecules in the MC to identify the best biomarker to predict the prognosis and identify potential hub genes. The final prediction model consisting of four cuprotosis molecules (LIAS, PDHA1, DLD and FDX1) showed that risk scores were significantly different among the three cuprotosis subtypes, with the highest score in Cluster B (Fig. 5A and B). According to the optimal cut-off value (1.02), all samples were assigned to a high or low-risk score group. Notably, the high-risk group had shorter survival than the low-risk group for all patients and GSE62254, GSE84437 and TCGA-STAD cohorts (Fig. 5C and Fig. S6A). Similarly, the high-risk group had more dead samples than the low-risk group among the abovementioned cohorts (Fig. S6B); risk scores were higher in dead patients than in alive patients (Fig. S6C). Consistent with these findings, Clusters B and C with high-risk scores were associated with short survival (Fig. 5 D).

Further, infiltration of B cell, CD4^+^ T cell, CD8^+^ T cell, dendritic cell, natural killer cell, neutrophil, regulatory T cell, T follicular helper cell and macrophages assessed by MCPCOUNTER, QUANTISEQ, CIBERSORT, XCELL, TIMER and EPIC were significantly and positively related to the risk scores of the cuprotosis signature (Fig. 5E). Based on the representative TCGA pathology slide, the high-risk score group was infiltrated by abundant immune cell compared to the low-risk score group (Fig. 5F). Moreover, the immune activation group had higher risk scores than the T-cell suppressive group, which was classified by Zeng D et al. [43] (Fig. 5G). There were significantly higher expressions of seven immune checkpoint genes, including DCD1LG2, TLR4, BTK, DYSF, LY96, CD44 and CD14, in the high-risk group (Fig. S6D). We also correlated risk scores with those genes scores of critical biological pathways of GC, and found that the cuprotosis signature was positively correlated with EMT1, angiogenesis, nucleotide excision repair, Wnt signalling pathway, antigen processing and presentation, TGF beta signalling pathway, immune checkpoint and DNA damage repair. On the other hand, it was negatively correlated with EMT2, EMT3, Pan F TBRS, mismatch repair, NF kappa B signalling pathway, ECM receptor interaction, JAK STAT signalling pathway, MAPK signalling pathway, PI3K Akt signalling pathway and CD8 T effector (Fig. S6E).

The GSE62254 cohort was used to explore the relationship of the risk scores of the cuprotosis signature with other subtypes of GC and clinical features. The risk score distribution was significantly different among AJCC stages, Lauren subtypes, MSI status and ACRG subtypes (Fig. 5H). Higher risk scores were found in the AJCC stage III and IV, MSS and EMT subtypes, all of which had poorer outcomes (Fig. S7A) [23,44].

### 3.6 Interaction between the cuprotosis signature and TMB on survival

We used the TCGA-STAD cohort to assess gene mutations of different risk score groups. It was found that missense variation had the highest frequency for both risk score groups (Fig. S7B). Compared to the high-risk score group, the low-risk group had more mutation counts (Fig. S7C). Moreover, there was a significant negative correlation between TMB and risk scores (Fig. S7D). Notably, TMB did not only have a significant association with better survival of GC, but also TMB and risk scores had a strong interaction effect on survival, with the poorest survival observed for the combination of low TMB group and high score group (Figs. S7E and F).

### 3.7 Hub genes of the cuprotosis signature were associated with survival and immunity of GC

**Fig. 6:**
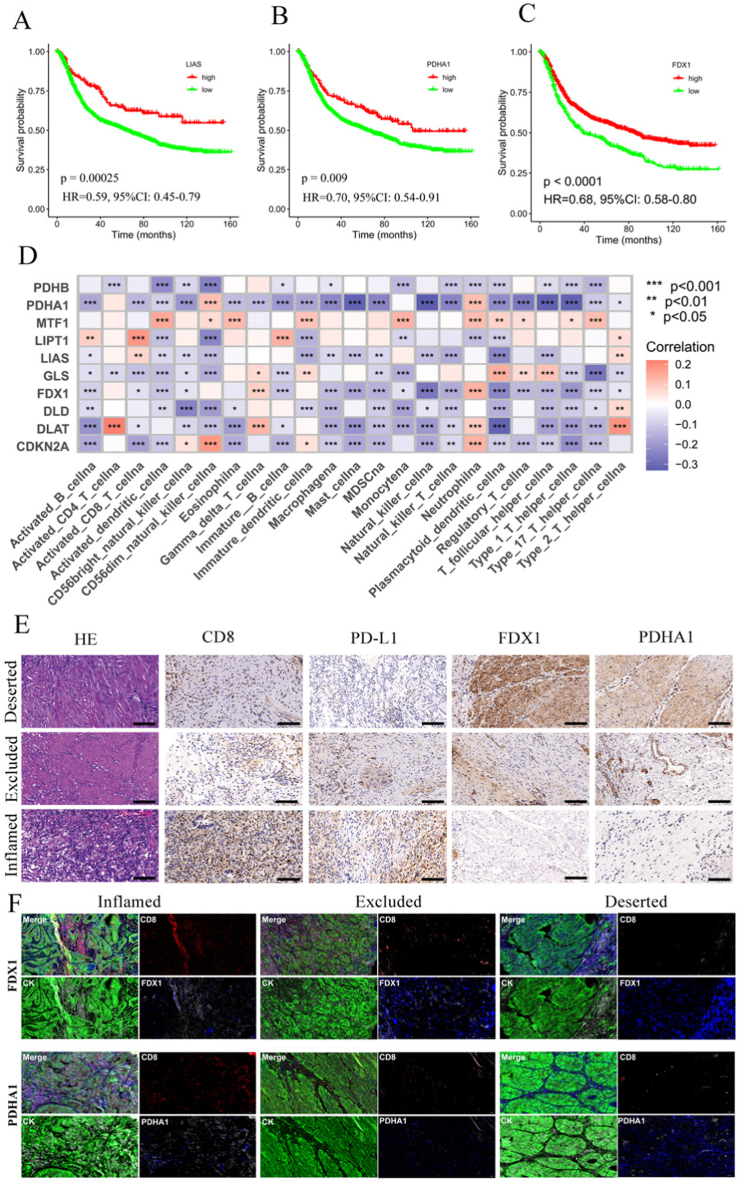
**LIAS, PDHA1 and FDX1 were correlated with tumor immune microenvironment. A-C** Relationships of LIAS, PDHA1 and FDX1 with the prognosis in 1544 GC patients. **D** Correlations between 10 cuprotosis molecules and immune cell infiltration. Spearman correlation analysis was performed. **E** Immunohistochemistry showed the expression of CD8, PD-L1, FDX1 and PDHA1 in tumor tissues. In three immunophenotypes, representative co-stained images of CD8, PD-L1, FDX1 and PDHA1. According to the spatial distribution of CD8^+^ T cells, tumor tissues were divided into three immunophenotypes, immune-inflamed, immune-excluded, and immune-deserted. The scale of 50 μm. **F** Multi-color fluorescence staining showed the expression of CD8, PD-L1, FDX1 and PDHA1 in tumor tissues. Representative co-stained images of CD8, PD-L1, FDX1 and PDHA1 in the three immunophenotypes. The scale of 50 μm. (For interpretation of the references to color in this figure legend, the reader is referred to the Web version of this article.)

According to the cut-off thresholds derived using the Youden index, the 10 cuprotosis gene expressions of 1544 samples as the continuous variables were regrouped into dichotomous variables (high-risk group and low-risk group). The log-rank test showed that LIAS, PDHA1, FDX1, DLAT, DLD, LIPT1 and MTF1 may be protective factors for 1544 individuals with GC, and CDKN2A and PDHB may be risk factors (Fig. 6A–C and Fig. S8A). The expression of most cuprotosis genes was negatively correlated with immune cells infiltrating the immune microenvironment of GC (Fig. 6D). We chose 25 FFPE samples and used HE staining, immunohistochemistry, and multi-color immunofluorescence to determine a potential relation between cuprotosis molecules and immunotherapy response in varying immune conditions. Next, CD8 cell infiltration in the tissues was used to classify GC subtypes into three immune subtypes: immune-inflamed, immune-excluded, and immune-deserted (Fig. 6E). CD8 and PD-L1 expression was found to be highest in the immune-inflamed subtype, while FDX1 and PDHA1 expression was found to be highest in the immune-deserted subtype (Fig. 6E).

In addition, we utilized a multi-color fluorescence staining methodology to examine the spatial interaction between the two genes and CD8 in distinct GC immune subtypes (Fig. 6F). Low levels of FDX1 and PDHA1 expression were observed in tumor tissues that had high levels of CD8 infiltration. There were positive and negative correlations of FDX1 and PDHA1 with CD8^+^ T cell infiltration found in pan-cancers, while there were two negative correlations for TCGA-STAD (Fig. S8B). These findings highlight two genes as potential moderators of CD8^+^ T cell function under GC immunotherapy.

### 3.8 Cuprotosis signature and critical biological pathways of GC

**Fig. 7:**
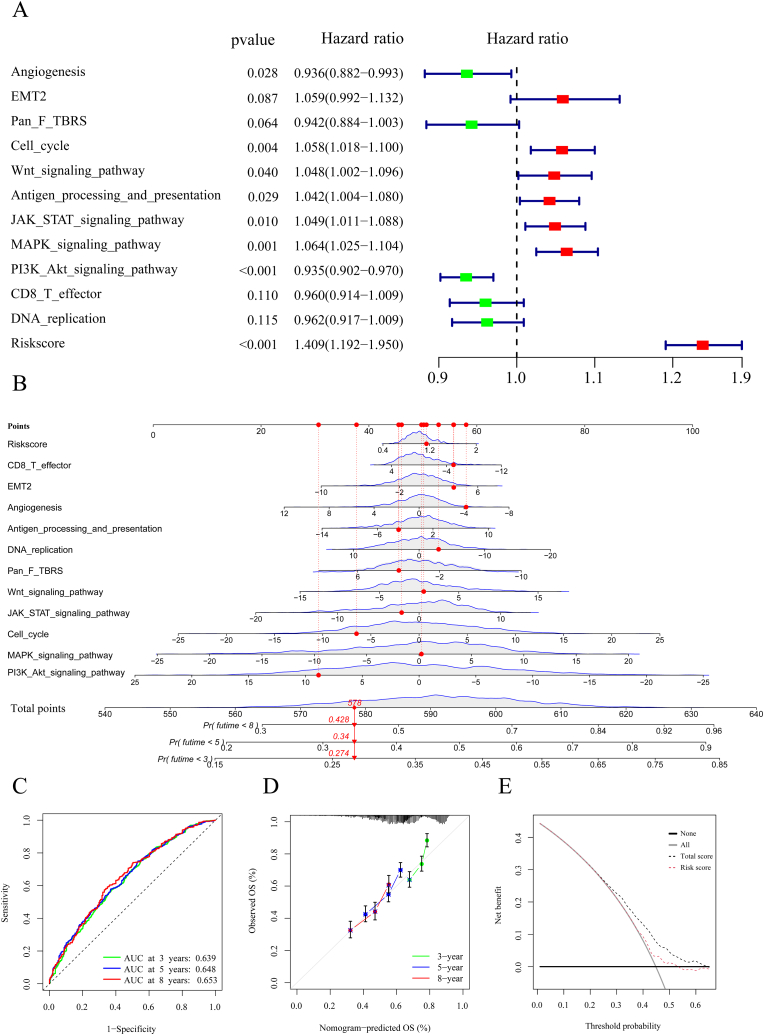
**The effect of the risk scores of the cuprotosis signature and critical signaling pathway scores on survival in GC. A** Multivariate Cox analysis of the cuprotosis signature and critical signal pathway. **B** Nomogram for predicting the three-, five- and eight-year survival of GC patients. **C** ROC curves for predicting three-, five- and eight-year survival. **D** Calibration curves for predicting the nomogram's three-, five- and eight-year survival. **E** Decision curve analysis of overall survival for the predicted nomogram model. Total score: the cuprotosis signature and critical signal pathway scores.

Univariate and multivariate Cox regression were used to screen potential influencing factors to further explore the effects of the cuprotosis signature and other key tumour-affecting pathways on survival. We used PCA to calculate the score of each pathway based on some marker gene expressions [45,46]. There were 22 pathways and risk scores of the cuprotosis signature associated with GC survival (Fig. S9). From the multivariate Cox regression for the above variables, it was found that angiogenesis, Pan F TBRS, PI3K Akt signalling pathway, CD8 T effector and DNA replication may be preventive factors of GC (all p < 0.12; Fig. 7A); whereas, EMT2, cell cycle, Wnt signalling pathway, antigen processing and presentation, JAK-STAT signalling pathway, MAPK signalling pathway and risk score were found to be risk factors. Using the results of a multivariate Cox regression analysis, a nomogram of the relevant factors was constructed to provide clinicians with a basis for predicting the prognosis of GC patients (Fig. 7B). From the nomogram, the three-, five- and eight-year survival rates of a representative sample (red dot; randomly selected patient No. 400) were 42.8%, 34.0% and 27.4%, respectively, according to total scores of each pathway and risk score. In addition, ROCs and calibration plots demonstrated that the nomogram was highly predictive of GC outcome (Fig. 7C and D). In contrast to only the cuprotosis signature (risk score), the combination of the cuprotosis signature and other key tumour-affecting pathways can have a high utility potential in decision making (Fig. 7E).

### 3.9 Immunotherapy response of the cuprotosis signature

The TIDE web server was used to predict the effect of the cuprotosis signature on the immunotherapy response based on standardised expressions of all genes in the MC. It was seen that risk scores of the cuprotosis signature were significantly positively related to CD8, Merck18, TIDE, Dysfunction, Exclusion and CAF and significantly negatively related to TAM M2, MDSC and MSI Expr Sig (Fig. S10A). Furthermore, the greater TIDE observed in the high-risk group lent a poorer response to immunotherapy in GC (Fig. S10B). Similarly, higher Dysfunction and Exclusion scores and lower MSI scores were found in the high-risk group (Fig. S10B).

Subsequently, we used the Kim cohort treated with PD-1 blocking agent to explore the immunotherapy response of the cuprotosis signature in GC. The prediction model of 10 cuprotosis molecules constructed by multivariate logistic regression showed impeccable performance in predicting immunotherapy response (Fig. S10C), without significant differences from the cuprotosis signature model constructed using LIAS, DLD, PDHA1 and FDX1 (Z = −1.36, P = 0.174). Therefore, we further selected the risk score of the cuprotosis signature to correlate with immunotherapy response and other clinical features. In addition, we discovered that the immunotherapy response, TCGA, and MSI subtypes all had very different distributions of risk scores (Fig. S10D). This result indicates the importance of the cuprotosis signature and provides the basis for performing a relationship analysis between cuprotosis molecules and immunotherapy.

## 4 Discussion

Cancer is a heterogeneous disease characterised by dysregulation of cell death [47]. Cuprotosis is a new cell death pattern that depends on copper-based and mitochondrial metabolic disorders. Research on targeted drugs regulating the cuprotosis pathway has provided a new idea for treating refractory cancer [48,49]. Therefore, there is an urgent need to evaluate the prognosis of GC and select potential therapeutic targets using cuprotosis as a basis. This study evaluated the prognosis, immune microenvironment, molecular characteristics, and immunotherapy response of three GC subtypes (Clusters A, B and C) using 10 cuprotosis molecules. Then, a risk score of the cuprotosis signature was proposed, which could quantify the prognosis and the number and type of immune cell infiltration in each sample. It was observed that the immune environment of GC and its impact on immunotherapy can be comprehensively understood from the perspective of cuprotosis to provide a reference for a follow-up personalised diagnosis and treatment plan.

Cell death is a conserved phenomenon in both prokaryotic and eukaryotic cells. First, cell death is divided into accidental and programmed cell death (PCD) based on morphological changes and DNA fragmentation [50]. PCD is considered a strict regulatory cell death (RCD) that occurs under physiological conditions. RCD can be mediated by a group of evolutionarily conserved pathways that play an essential role in developing immune response [51]. In general, the induction and execution of RCD are regulated by forming signal amplification complexes. Previous studies have suggested that cuprotosis is copper-dependent, modulated and distinct from other known mechanisms of cell death regulation [4].In addition, several types of RCD, including apoptosis, pyroptosis, necroptosis and ferroptosis, have been widely studied for the oncogenesis, progression, metastasis and immunotherapy of different types of cancers [52,53]. The uncontrolled death of single or mixed RCDs can lead to various human diseases, including cancer. The uncontrolled death of single or mixed RCDs can lead to various human diseases, including cancer. Moreover, several cancers, including pancreatic adenocarcinoma, colon cancer, ovarian cancer, liver cancer, triple-negative breast cancer, bladder cancer, and clear-cell renal cell carcinoma, may benefit from the utilization of cuproptosis-related genes in prognosis prediction, immune response evaluation, and tumor classification [[54], [55], [56], [57], [58], [59], [60]]. We also established a risk score based on the cuprotosis signature that was able to predict the prognosis, response to ICIs and GC subtype of patients.

Copper is a fundamental element in sustaining human life and plays an essential role as a cofactor of essential enzymes [61]. Under normal conditions, the intracellular copper ion concentration is kept at a low level through an active homeostasis mechanism. When the copper ion level accumulates beyond the threshold, the excess copper ion leads to excessive cell respiration, resulting in cytotoxicity and, ultimately, death. Notably, cancer cells have a higher demand for copper than normal cells [62]. Some cancers express large amounts of thiooctyliated mitochondrial proteins and exhibit high intensity of respiration [49]. Similarly, in this study, the differentially expressed genes among three subtypes of cuprotosis were enriched in key signalling pathways for cell death, including the citrate cycle (TCA cycle), cell cycle and p53 signalling pathway. Moreover, elevated copper concentrations have been found in animal models and in tumour tissue or serum of patients with various cancers [63,64]. Therefore, a copper-chelating agent should be developed as an adjuvant therapy for tumours.

We found that seven of the 10 genes that promote copper-induced cell death – LIAS, FDX1, LIPT1, DLD, PDHA1, DLAT and PDHB – were down-expressed. At the cellular level, it has been confirmed that the knockout of the above genes can inhibit the death of ABC1 and OVISE cells induced by CuCl_2_ and elesclomol [4]. This further explains the mechanism of copper death in cells. Their low expression limits the aggregation of the key proteins – mitochondrial respiratory protein and lipoacylation protein (DLAT, DLST). This aggregation can lead to protein toxic stress and eventually cell death. In Fig. 2D, the expression correlation of the above seven key genes promoting copper-induced cell death is positive, which also shows the consistency of these genes in mitochondrial metabolic function and their common role in regulating cuprotosis. Interestingly, CDKN2A, a well-known tumour suppressor gene, is highly expressed in pan-cancer types. CDKN2A encodes two proteins, p16 (INK4) and P14 (ARF), which regulate CDK4 and p53 to control the transition of cells from the G1 phase to S phase [65]. Gene mutation or deletion is related to a variety of tumours. A previous study showed that CDKN2A deletion could lead to hematogenous metastasis of GC [65]. One possible mechanism is that CDKN2A inhibits cell in cell (CIC) structures to limit the crosstalk between the multiple signals of each and thus decreases cancer cell death [66].

This study aimed to use 10 cuprotosis molecules from 1544 individuals to classify GC and further assess the characteristics of the immune microenvironment and prognosis in each subtype. We found a negative correlation between CNV and mRNA in most pan-cancer types. It is well known that tumours are a progressive disease. A series of genomic molecular changes will occur in the process of tumorigenesis and development. CNV of genes is generally considered an important source of genome sequence differences among individuals [67]. CNV is not only associated with benign gene polymorphisms, but also with malignant diseases [68]. Gene CNV in protein coding genes and regulatory regions can lead to changes in gene expression and is associated with a variety of tumours [69]. In GC, except for CDKN2A, the CNV and mRNA of the other nine genes are negatively correlated, indicating that individuals with the above gene mutations may experience copper-induced cell death, which provides a reference for screening the population who have been treated for copper death.

In this study, the cuprotosis genes selected included seven genes (LIAS, LIPT1, DLD, DLAT, PDHA1, PDHB and FDX1) positively regulating and three genes (MTF1, GLS and CDKN2A) negatively regulating the copper death metabolic pathway. Survival analysis showed that LIAS, FDX1, and PDHA1 might be preventive factors of survival in the MC. Similarly, abnormal expression of LIAS in lung tumour tissues may induce altered signal transduction pathways that are beneficial to cell survival and decrease the overall intracellular oxidation state, promoting survival or anti-apoptotic effects [70]. FDX1 impacted the prognosis of lung adenocarcinoma and was closely related to glucose metabolism, fatty acid oxidation and amino acid metabolism [71]. Decreased PDHA1 expression was associated with poor overall survival of individuals with oesophageal squamous cell carcinoma, prostate cancer, ovarian carcinoma, and GC [[72], [73], [74], [75]]. Likewise, in this study, the risk score of four cuprotosis genes, LIAS, FDX1, PDHA1 and DLD, could predict GC prognosis. Based on these results, the stratification of GC by these four cuprotosis genes and their constructed risk scores may be a new direction for research on GC therapy.

In the study, cuprotosis has a wide-ranging regulatory mechanism that affects the tumor-immune microenvironment, clinicopathological characteristics, and prognosis. We also analyzed cuprotosis for its potential as a targeted or immunotherapeutic intervention. These findings emphasize the importance of cuprotosis in the clinical diagnosis and treatment of GC, and provide a foundation for selecting prospective clinical targets and identifying particular patients for individualized treatment.

### 4.1 Limitations and clinical translation of the study

Several limitations exist in the current study. To begin, all analyses were performed only on information gathered from freely available sources. To further validate its clinical utility, prospective real-world data are needed. Second, more in vivo and in vitro experimental research are required to determine the molecular mechanisms processes by which 10 cuprotosis molecules affect the immune microenvironment in GC tissues. Third, most datasets included information on a variety of important clinical events that may have altered the prognosis of the immune response and cuprotosis status, such as chemoradiation, neoadjuvant chemotherapy, and surgery.

The cuprotosis as a new cell death mode, promoting antitumor therapy targeting copper may be an effective therapeutic strategy. First, a copper complexing agent binds copper ions and lessens their concentration, which blocks tumor cells from proliferating and metastasizing [76]. Second, copper ions are introduced into cells using copper ionophores to raise intracellular Cu2+ concentration, induce reactive oxygen species, and trigger tumor cell death [77]. The cuprotosis in the clinical management for patients who have PD-1 resistance to immunotherapy might be a useful option [78]. Especially, copper nanoparticles with nano scale, outstanding biocompatibility offer a wide range of potential uses for cuprotosis in cancer treatment.

## Ethics approval and consent to participate

The patient data in this work were acquired from the publicly available datasets whose patients’ informed consent was complete. Other data are available upon reasonable request. 25 GC patients were selected in the clinical dataset. Written informed consent was obtained from each participant.

## Author contribution statement

Ke-wei Wang and Mei-dan Wang: Conceived and designed the experiments; Performed the experiments; Analyzed and interpreted the data; Contributed reagents, materials, analysis tools or data; Wrote the paper.Zi-xi Li, Ben-shun Hu, Jian-feng Huang and Jun-jie Wu: Performed the experiments; Analyzed and interpreted the data; Contributed reagents, materials, analysis tools or data.Zheng-dong Yuan: Analyzed and interpreted the data; Performed the experiments; Wrote the paper.Xiao-long Wu and Qin-fang Yuan: Analyzed and interpreted the data; Contributed reagents, materials, analysis tools or data.Yi-fan Sun and Feng-lai Yuan: Conceived and designed the experiments; Analyzed and interpreted the data; Wrote the paper.

## Funding statement

This work was supported by the top Talent Support Program for young and middle-aged people of Wuxi Health Committee [HB2020040], 10.13039/501100016308Mega-project of Wuxi Commission of Health [Z202007], Taihu Zhiguang Science and Technology Project [Y20212015].

## Data availability statement

Data will be made available on request.

## Declaration of competing interest

The authors declare that they have no known competing financial interests or personal relationships that could have appeared to influence the work reported in this paper.

## Appendix A Supplementary data

Multimedia component 10 Multimedia component 10

figs1 figs1

figs2 figs2

figs3 figs3

figs4 figs4

figs5 figs5

figs6 figs6

figs7 figs7

figs8 figs8

figs9 figs9

figs10 figs10
