# Supplementary figures and images for: Systematic analysis of the cuprotosis in tumor microenvironment and prognosis of gastric cancer
Source: Heliyon. 2023 Feb 17;9(3):e13831. doi: 10.1016/j.heliyon.2023.e13831 (PMC9988515; doi:10.1016/j.heliyon.2023.e13831)

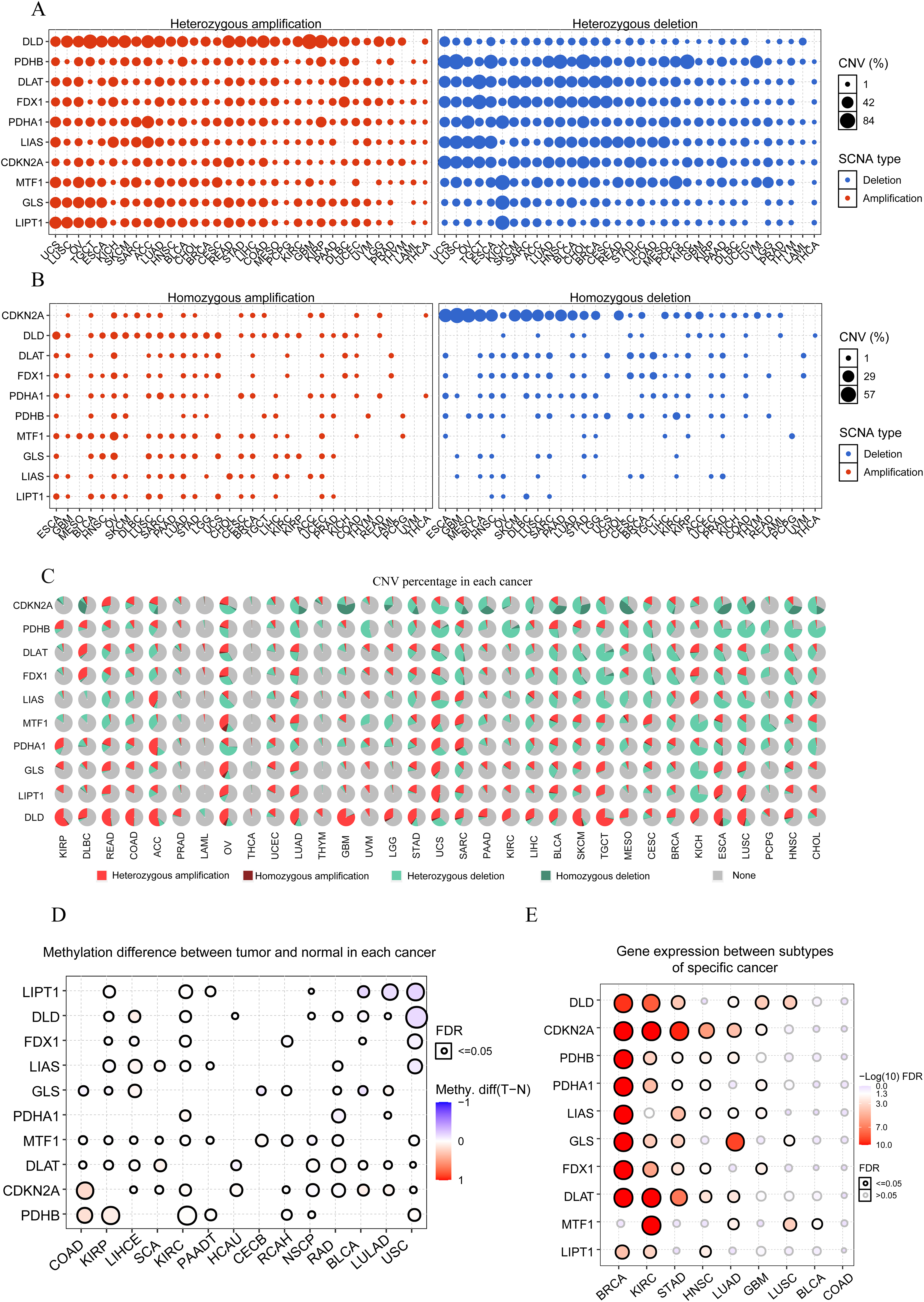

Supplement: figs1 [file mmcfigs1.jpg]

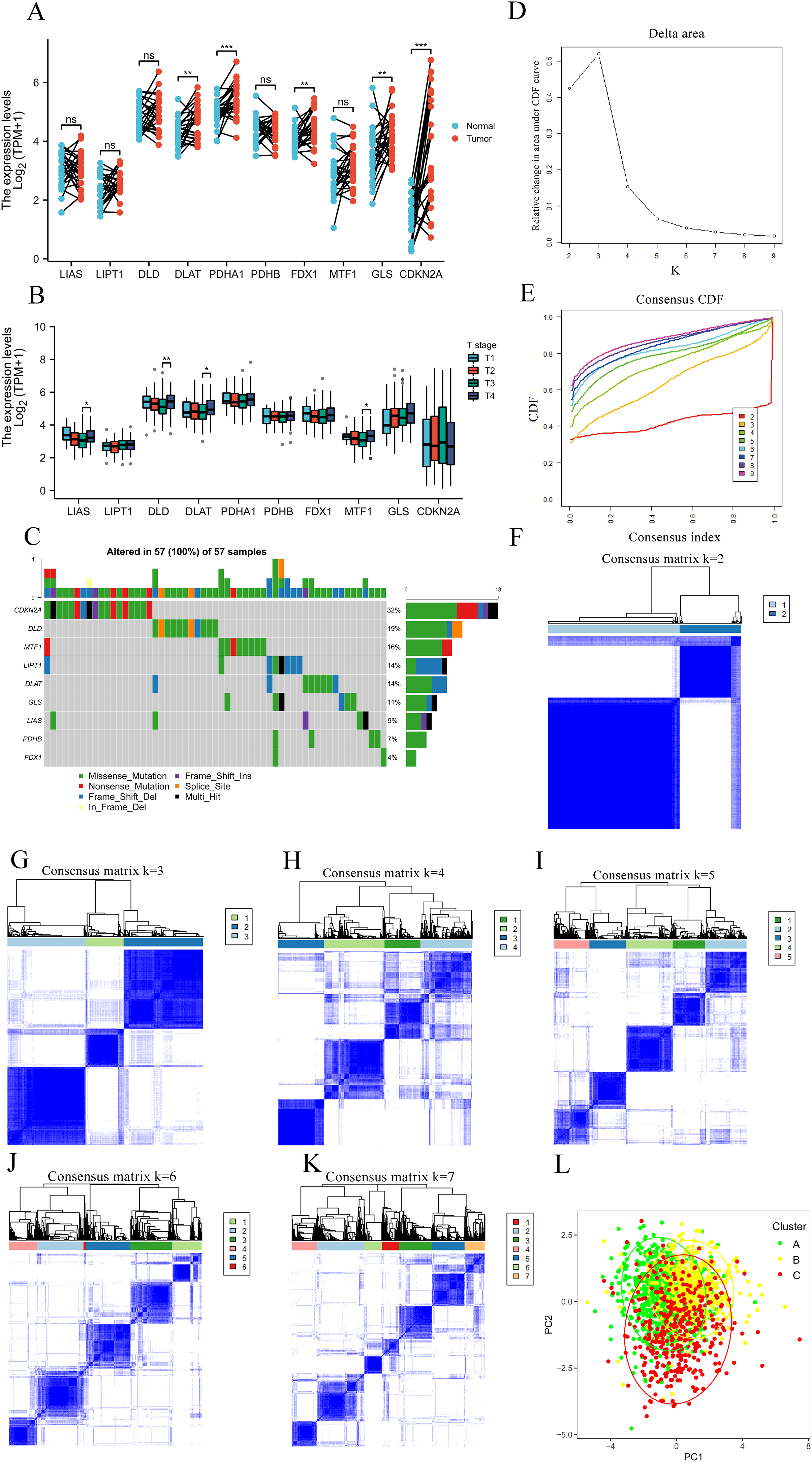

Supplement: figs2 [file mmcfigs2.jpg]

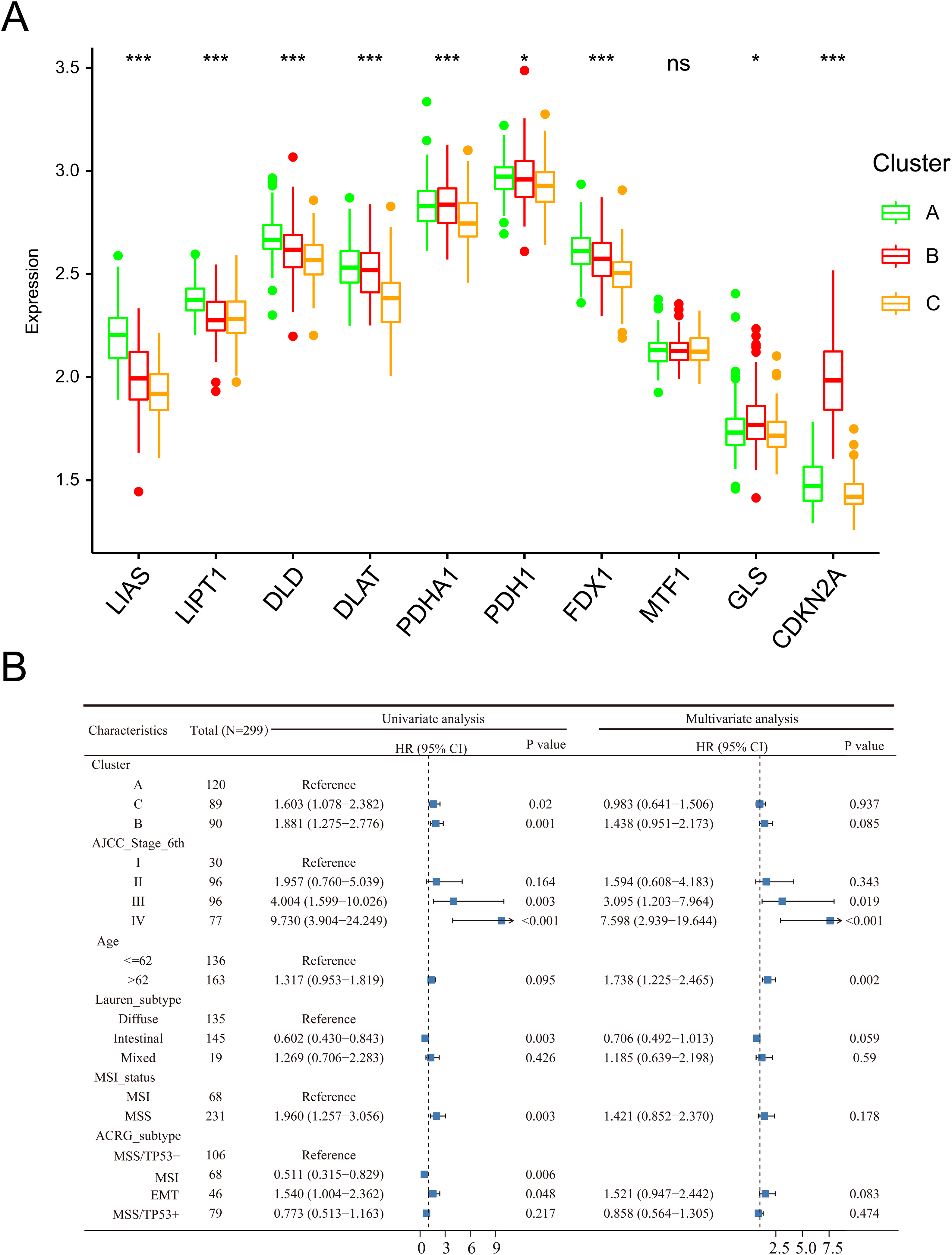

Supplement: figs3 [file mmcfigs3.jpg]

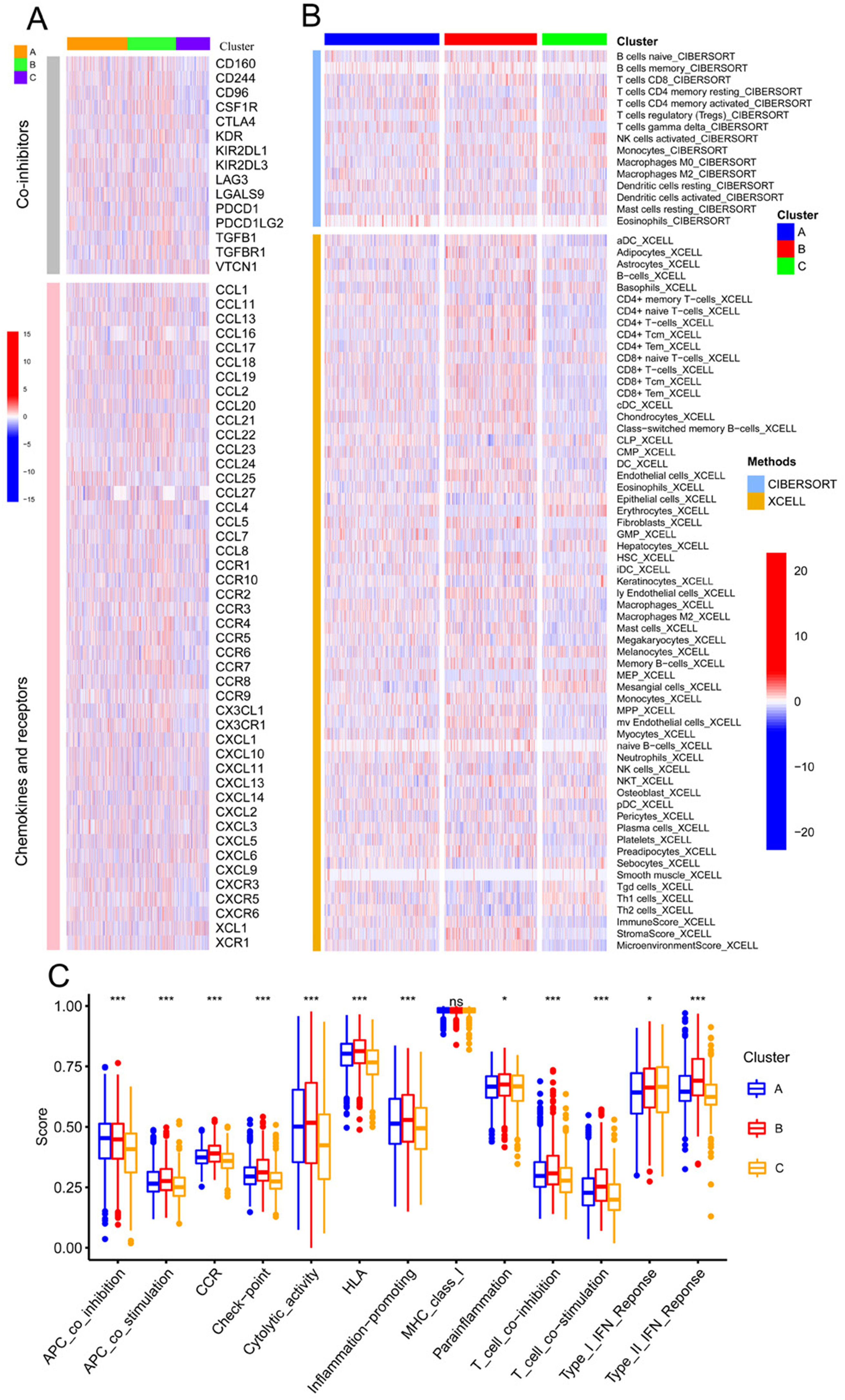

Supplement: figs4 [file mmcfigs4.jpg]

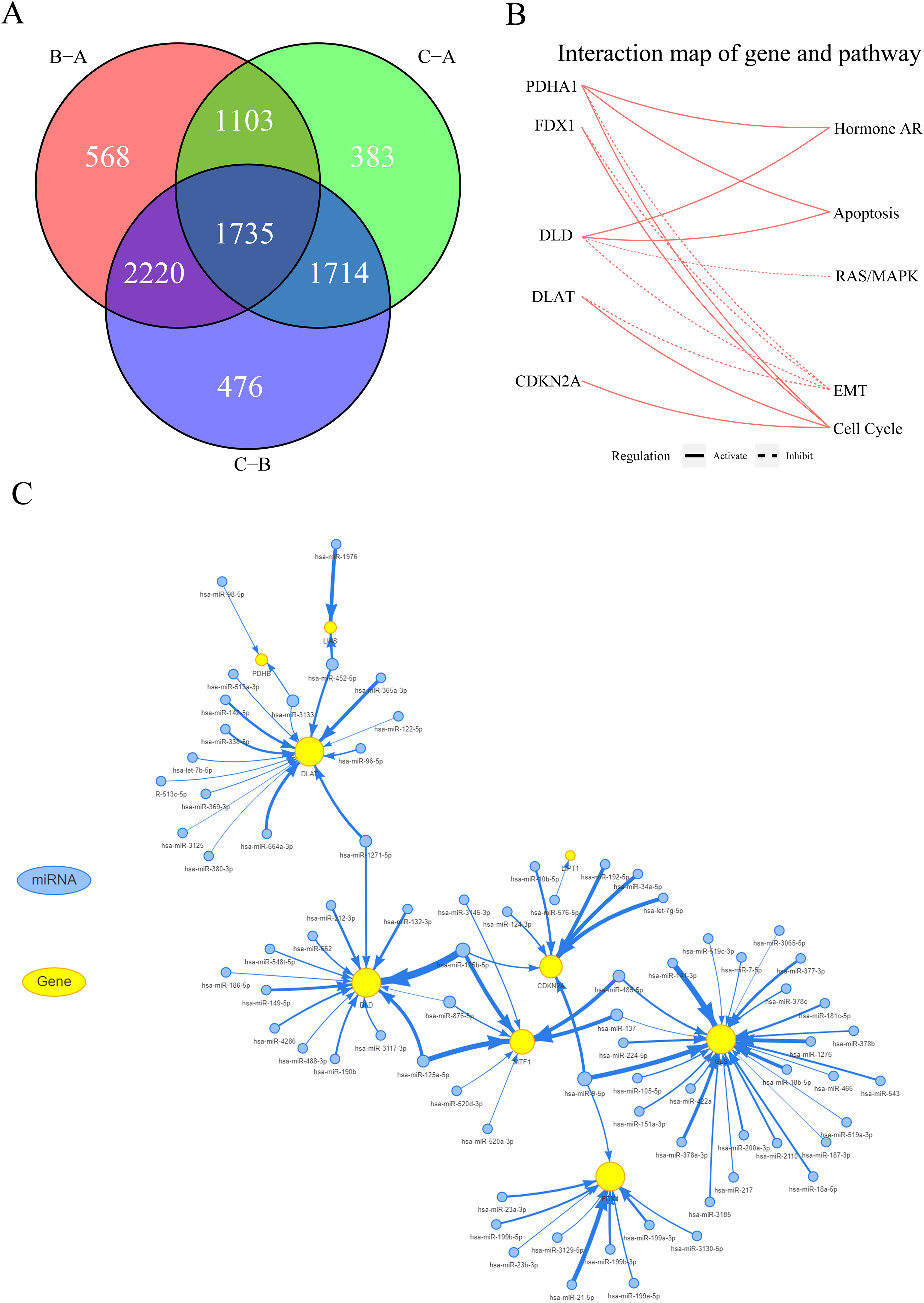

Supplement: figs5 [file mmcfigs5.jpg]

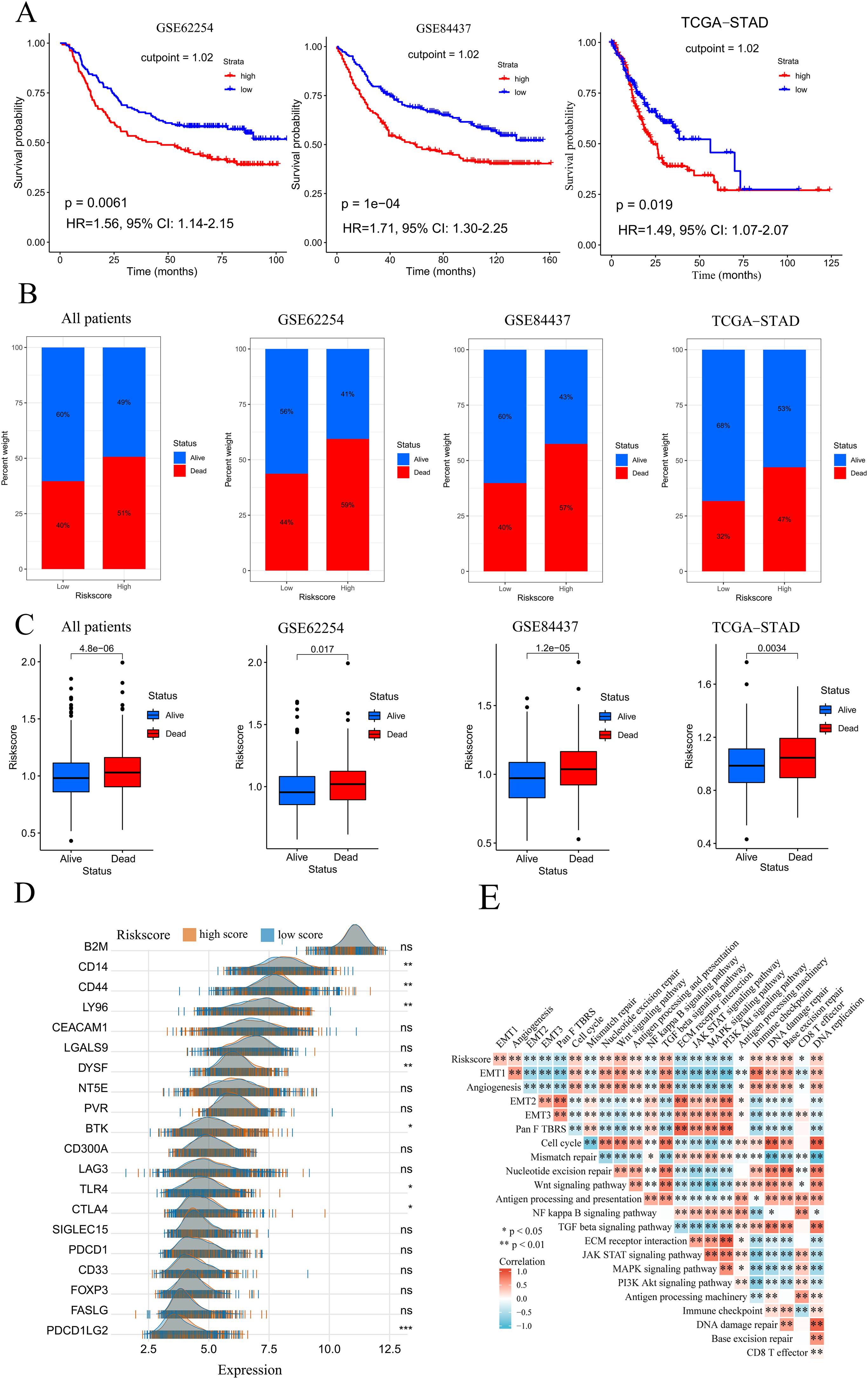

Supplement: figs6 [file mmcfigs6.jpg]

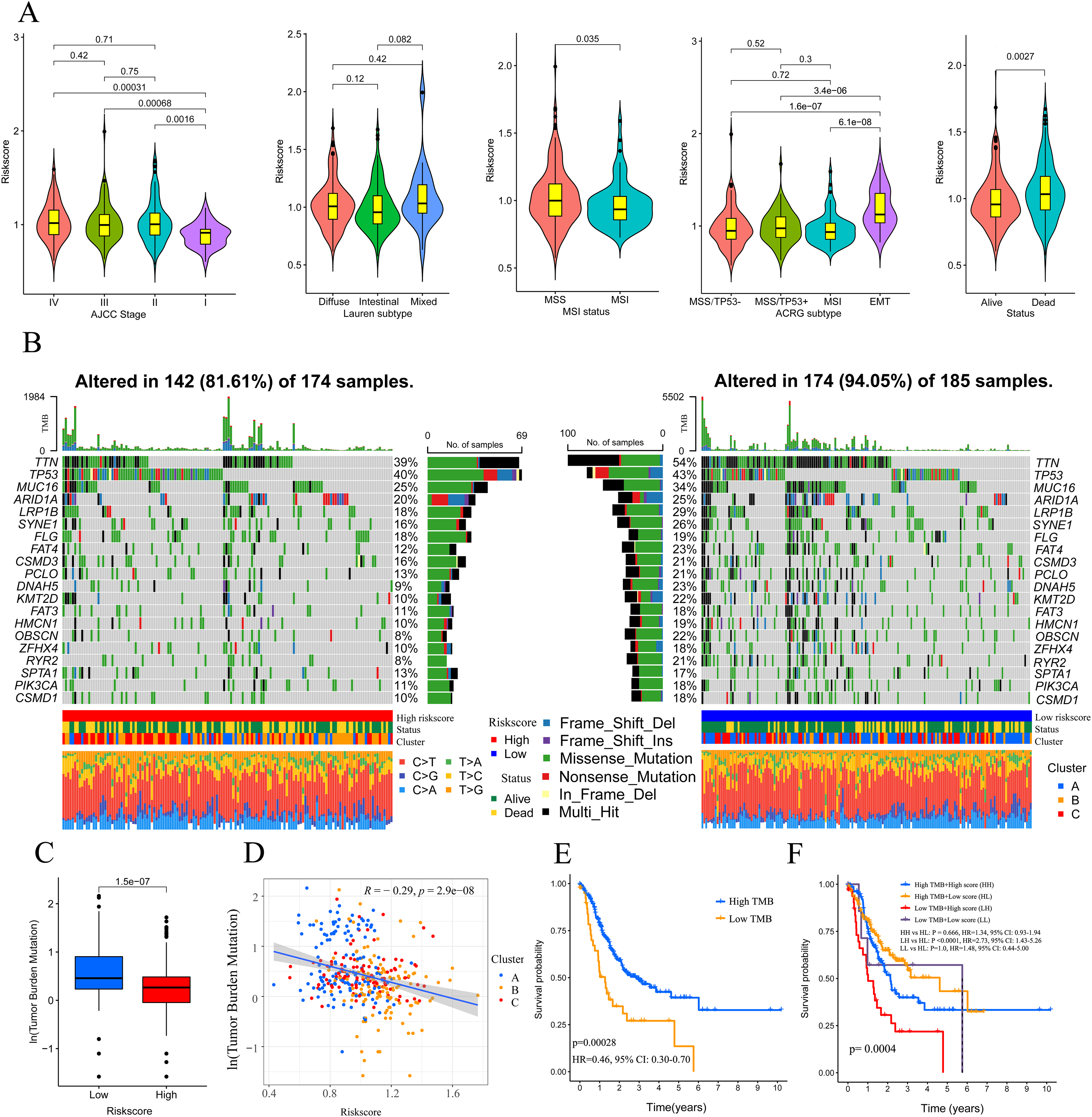

Supplement: figs7 [file mmcfigs7.jpg]

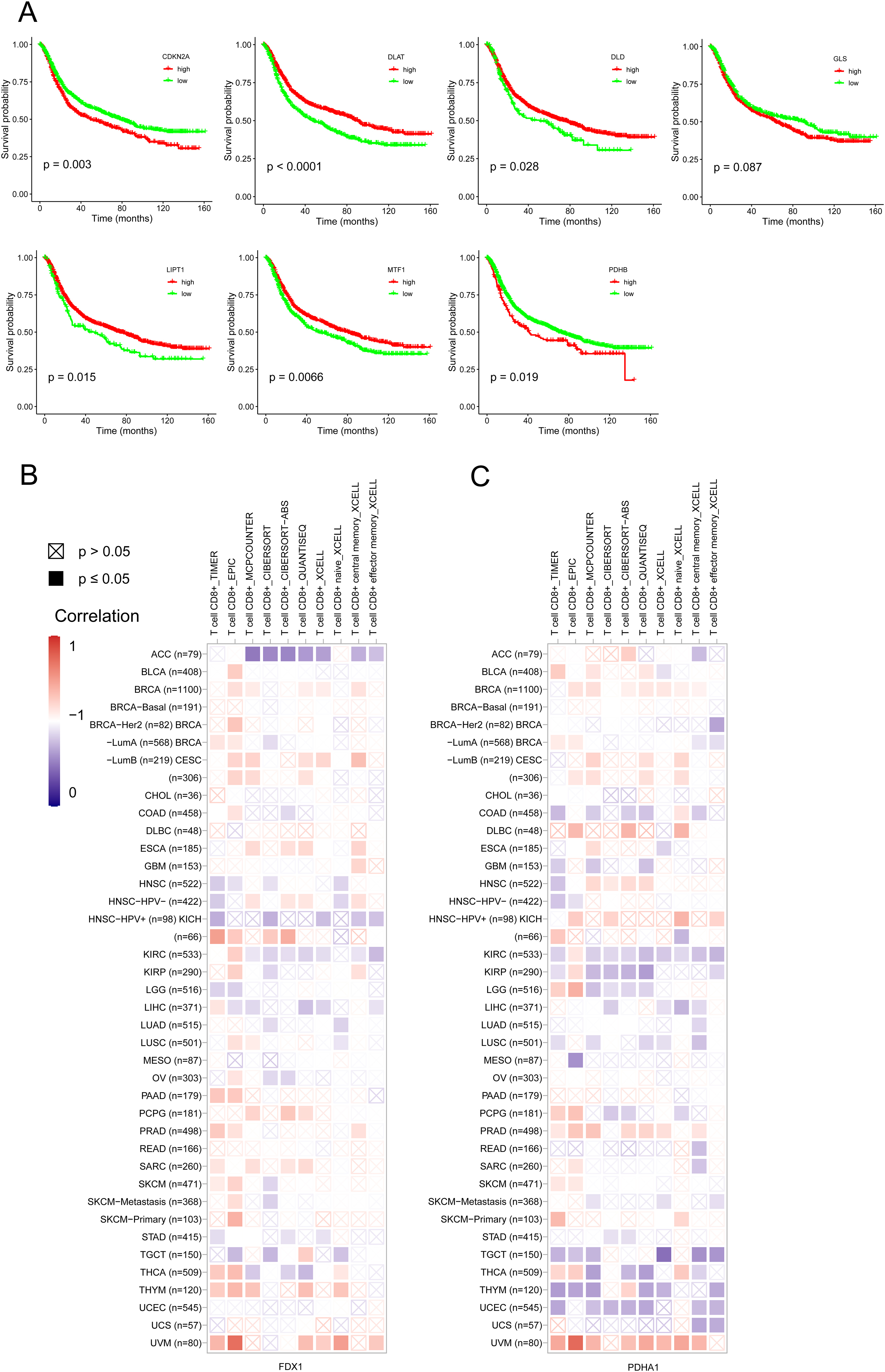

Supplement: figs8 [file mmcfigs8.jpg]

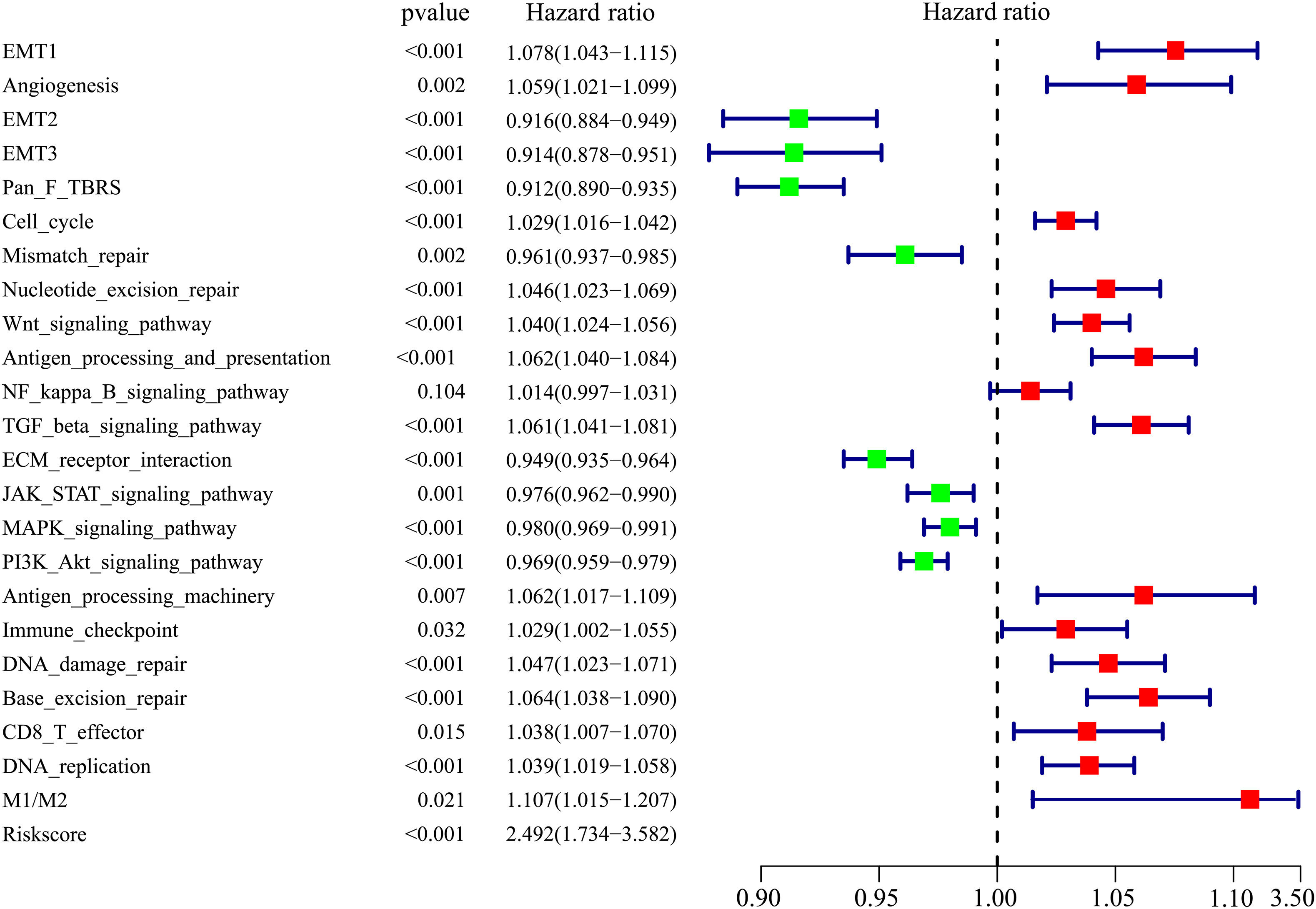

Supplement: figs9 [file mmcfigs9.jpg]

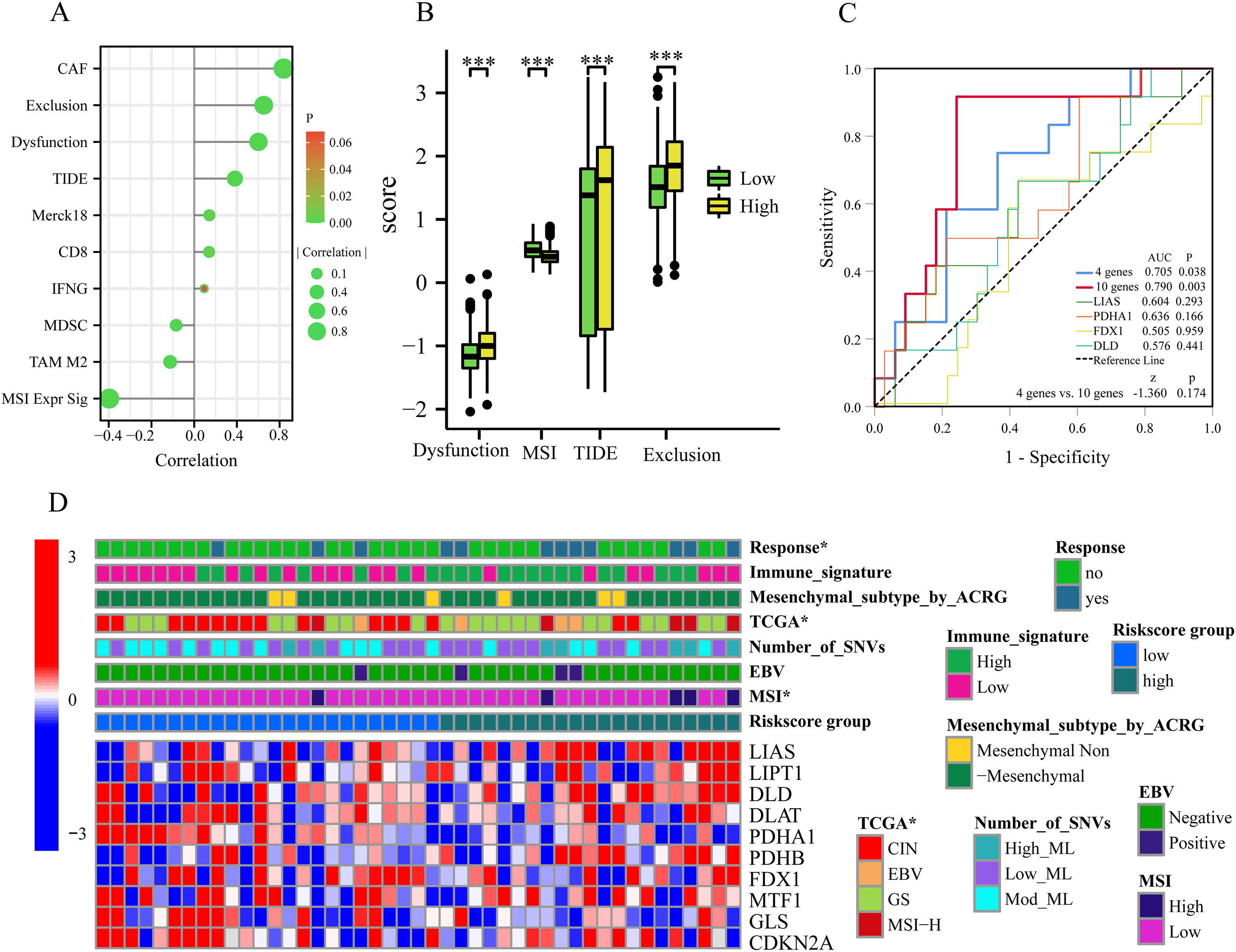

Supplement: figs10 [file mmcfigs10.jpg]
